# Are We Adequately Assessing Delirium? An Analysis Of Liaison Psychiatry Referrals

**DOI:** 10.1192/j.eurpsy.2023.1101

**Published:** 2023-07-19

**Authors:** E. Tripp, M. Aremu Falade, M. Alves, M. Davies, J. H. Tan, L. Premalatha

**Affiliations:** Croydon Liaison Psychiatry, Croydon University Hospital, London, United Kingdom

## Abstract

**Introduction:**

Delirium is characterised by an acute, fluctuating change in cognition, attention and awareness (Wilson et al. Nature Reviews 2020; 6). This presentation can make the diagnosis of delirium extremely challenging to clinicians (Gofton., Canadian Journal of neurological sciences. 2011; 38 673-680). It is commonly reported in hospitalised patients, particularly in those over the age of sixty five (NICE. Delirium: prevention, diagnosis and management. 2010).

**Objectives:**

Our aim is to identify which investigations and cognitive assessments are completed prior to a referral to the liaison psychiatry services in patients with symptoms of delirium.

**Methods:**

Referrals (N = 6012) to the liaison psychiatry team at Croydon University Hospital made between April and September 2022 were screened. Search parameters used to identify referrals related to a potential diagnosis of delirium were selected by the authors. The terms used were confusion; delirium; agitation; aggression; cognitive decline or impairment; disorientation; challenging behaviour. Data was collected on the completion rates of investigations for delirium as advised by the NICE clinical knowledge summaries. Further data was gathered on neuroimaging (CT or MRI), cognitive assessment tools (MOCA/MMSE) and delirium screening tools (4AT/AMTS).

**Results:**

The study sample identified 114 referrals (61 males and 53 females), with 82% over 65 years at the time of referral. In 96% of referrals, U&E and CRP were performed. Sputum culture (1%), urine toxin screen (4%) and free T3/4 (8%) were the tests utilised the least. Neuroimaging was completed in 41% of referrals (see Graph 1 for a full breakdown of results).

A formal cognitive assessment or delirium screening tool was completed in 32% of referrals. The AMTS and 4AT tools were documented for 65% and 24% respectively. A total of 19 referrals explicitly stated the patient was suspected to have dementia. A delirium screening tool was documented in 47% of these cases however, a formal cognitive assessment was documented in only 5% of these patients.

Following psychiatric assessment 47% of referrals were confirmed as delirium.

**Image:**

**Figure:**
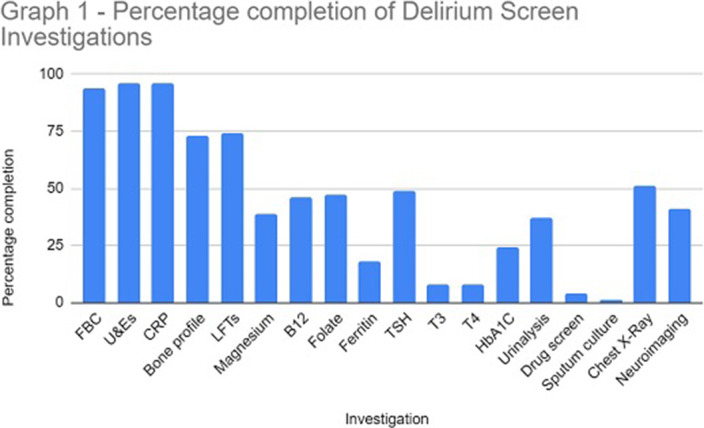

**Conclusions:**

Our data highlights the low level completion of the NICE recommended delirium screen prior to referral to liaison psychiatry. The effective implementation of a delirium screen and cognitive assessment is paramount to reduce the number of inappropriate psychiatric referrals in hospital and helps to identify reversible organic causes of delirium. This in turn will ensure timely treatment of reversible causes of delirium and reduce the length of hospital admission.

**Disclosure of Interest:**

None Declared

